# The effects of musical practice on the well-being, mental health and social support of student, amateur, and professional musicians in Canada during the COVID-19 pandemic

**DOI:** 10.3389/fpsyg.2024.1386229

**Published:** 2024-06-07

**Authors:** Audrey-Kristel Barbeau, Isabelle Héroux, Gina Ryan, Louis-Édouard Thouin-Poppe

**Affiliations:** ^1^Département de Musique, Faculté des Arts, Université du Québec à Montréal (UQAM), Montréal, QC, Canada; ^2^Faculté des Arts, Université du Québec à Montréal (UQAM), Montréal, QC, Canada

**Keywords:** COVID-19 in Canada, music-making, well-being, mental health, social support

## Abstract

This pan-Canadian study investigates the effects of musical practice on the well-being, mental health, and social support of Canadian musicians during the COVID-19 pandemic. Using a survey questionnaire, data was collected from 1,618 participants aged 14 and above during the first wave of the pandemic up to the first half of 2022. The survey included standardized questionnaires to self-assess well-being (WHO-5), mental health (MHC-SF), and social support (SPS-10 measures social support). Results show that increased musical practice frequency correlates with improved well-being and mental health, particularly among amateurs. Professional musicians and those at a post-secondary level exhibit lower well-being scores, likely due to pandemic-related challenges. Factors such as age, gender, sports engagement, and participation in social clubs or volunteer work significantly influenced outcomes. While sports engagement was associated with higher scores on well-being, mental health and social support, no significant differences were found among participants engaged in artistic hobbies. As for involvement in social clubs or volunteer work, benefits were reported on two of the three outcomes. Overall, the findings suggest that regular amateur musical practice, especially in group settings, alongside engagement in sports and social activities, may have promoted well-being, mental health, and social support among musicians during the challenging period of the COVID-19 pandemic.

## 1 Introduction

The pandemic impacted several aspects of our daily lives, and drastically changed how we engaged with each other. The period of the pandemic gave rise to a drastic increase in people’s levels of stress, anxiety and loneliness (Card et al., 2022), forcing governments to address rising public health issues related to mental health (Cost et al., 2022). The pandemic also impacted how we engaged in music practice. On the one hand, ensemble membership declined as group music-making either came to a halt or was offered in an online format during periods of confinement (Kearney et al., 2021). Conversely some studies show that, as a result of online options, the pandemic provided new opportunities for arts engagement (Mak et al., 2021) and that musical engagement increased among the general population (Cabedo-Mas et al., 2021; Ferreri et al., 2021). For example, one study found an increase in at-home musical behaviors of young children, such as singing and doing music, in Brazil (Ribeiro et al., 2021). In a separate study on 786 Canadian university students, Finnerty et al. (2021) found that students who indicated being more open to experience were more likely to be involved in musical activities, such as playing instruments, singing or song writing. A British study revealed that participation in virtual music groups offered significant psychological support for children and young people, helping them express themselves, manage emotions, regain confidence, and maintain social connections. While virtual group music-making indirectly fostered a sense of belonging, similar to in-person experiences, the direct connection observed in traditional settings was not replicated online (Levstek et al., 2021).

It is not surprising that several studies indicated an increase in music listening during the pandemic (Carlson et al., 2021; Vidas et al., 2021), including greater use of online listening platforms, such as Spotify and TikTok, which favor the discovery of new music (Hurwitz and Krumhansl, 2021). Hurwitz and Krumhansl (2021) reported that listening to music helped American university students to uplift their mood and alleviate sensations of loneliness and isolation. In a study on 402 university students in Australia, Vidas et al. (2021) found that music listening was a highly effective strategy for stress management.

Long before the Covid-19 pandemic, music has been recognized for its social and psychological benefits. Researchers have studied the positive effects of music on multiple populations, including students from preschool to high school and beyond (Harland et al., 2000; Hallam, 2010; Kokotsaki and Hallam, 2011), marginalized youth (Parker, 2018), adults (Coffman, 2002) and older adults (Creech et al., 2014). Participation in musical activities and ensembles has been shown to provide social support for both adults and school-aged children (Scripp, 2002; Carucci, 2012; Osborne et al., 2016) and several studies have shown that participation in choirs has a positive effect on well-being for both professional and amateur musicians (Judd and Pooley, 2014; Stewart and Lonsdale, 2016).

Canada boasts the world’s second-largest landmass (9,984,670 km^2^), but the country ranks only 38th in population (Bossé, 2023). Unevenly distributed between 10 provinces and three territories, its population is concentrated in southern regions and cities. From the 38,683,567 inhabitants in 2022, 1,807,250 were descendants of the First Nations who were the first to inhabit the territory (Government of Canada, 2024). Census data from 2021 indicate that 15% of the Canadian population considers itself to be of diverse ethnic and cultural origins (Statistics Canada, 2023b). While there are a variety of languages spoken by Canadians, French and English are the country’s two official languages. Since 1867, Canada has been constituted as a monarchy with parliamentary democracy (Marleau and Montpetit, 2000). In the Canadian federation, provinces and territories function as partially autonomous, particularly in the areas of health, education and cultural policies (Marleau and Montpetit, 2000).

As the Canadian population density varies from one area to the other and is spread over a vast territory where each province is responsible for its health system, sanitary measures during Covid-19 were implemented differently and within various timeframes. For example, the length of extreme lock-down procedures varied from cities to towns across the country (City of Toronto, 2020; Ontario – Office of the Premier, 2020). In addition, school closures were inconsistent across provinces and even in regions within provinces, with a range of 9 weeks in Quebec up to 19 weeks in Ontario (Gallagher-Mackay et al., 2021). The continuation of musical activities has varied significantly from one school to another, ranging from the temporary suspension of extracurricular activities to the cessation of all music classes (Boucher et al., 2022). For example, a study in Quebec showed that high schools were more affected than primary schools due to the fact that music classes in high schools are often taught in large ensembles, such as wind ensembles and orchestras (Barbeau et al., 2023).

*Statistics Canada* serves as the national statistical office, dedicated to providing Canadians with essential information about the country’s economy, society, and environment. Statistics are measured quarterly and the first and second quarters of 2022 (Statistics Canada, 2023d) correspond with the timeframe during which participants responded to our survey. Employing the Canadian Social Survey, Statistics Canada monitored mental health, well-being and sense of belonging of the Canadian population during COVID 19.

Mental health is defined by the World Health organization (WHO) as “a state of well-being in which the individual realizes his or her own abilities, can cope with the normal stresses of life, can work productively and fruitfully, and is able to make a contribution to his or her community” (World Health Organization, 2004, p. 2). The WHO emphasizes that there is a close relationship between positive mental health and well-being: “mental health is the foundation for well-being and effective functioning for an individual and for a community” (p. 2). The indication of “effective functioning for a community” suggests a link between mental health, well-being and social functioning, which seems aligned with Keyes’ tripartite model of well-being. Indeed, Keyes (2014) indicates that subjective well-being is a combination of emotional, psychological and social well-being, and can be linked to the perceived quality a person attributes to his or her life. Social well-being may thus be associated with social relationships, which may also be linked to perceived social support. Gottlieb and Bergen (2010) define social support as “the social resources that persons perceive to be available or that are actually provided to them by nonprofessionals in the context of both formal support groups and informal helping relationships” (p. 512). While Statistics Canada does not measure social support *per se*, it uses the concept of sense of belonging, which is considered by multiple authors (for instance, Cohen and Wills, 1985; Schonfeld, 1991; Suvak et al., 2013) as a dimension of social support. As such, the data provided by Statistics Canada may shed light on this dimension of social support, in addition to its data associated with mental health and well-being.

In the initial quarter of 2022, in response to the Statistics Canada query “In general, how is your mental health?,” participants rated it as excellent or very good at 47%, good at 33%, and fair or poor at 20%. During the second quarter, the figures remained similar, with the exception that 1% of respondents transitioned from a good rating to an excellent one. If we delve a bit further into the subgroups of respondents, we observe that men perceive their mental health more positively than women. For instance, in the first quarter, 57% of men consider their mental health excellent or very good, in contrast to 51% of women. Respondents identifying as LGBTQ2 + ^1^ rate their mental health lower than other subgroups, with 25.5% considering it excellent or very good, 25.1% rating it as good, and 49.3% indicating that their mental health is fair or poor (Statistics Canada, 2023c). A similar trend of men rating higher than women is observed within this subgroup. For instance, in the first quarter of 2022, 29.3% of LGBTQ2+ men considered their mental health excellent or very good, compared to 23% of women. Additionally, it is noted that the older the respondent group, the more positively their mental health is perceived. Thus, the 15–24 age group is the one with the lowest scores, with 39% of respondents rating their mental health as excellent or very good, 29% as good, and 32% as poor.

During the 2021–2022 timeframe, individuals in the 65 to 84 age group in Canada consistently reported the highest levels of perceived well-being (Statistics Canada, 2023e). This observation stems from an examination of several quality of life indicators from their comprehensive analysis. More precisely, 41% of individuals aged 65 to 84 attained a combined high score, contrasting with only 25% of individuals observed in the 20 to 29 age group. It is noteworthy that, regardless of age, both men and women consistently reported similar combined perceived well-being scores. For respondents identifying as LGBTQ2+, 18% reported having a high perceived well-being. Within the LGBTQ2+ community, the younger demographic, specifically youth and young adults aged 15 to 29 years, exhibited the lowest likelihood of highly perceived well-being at 10%. This situation is not specific to Canada. A literature review targeting adolescents from several countries (Garagiola et al., 2022) showed loneliness, depression and anxiety were experienced during pandemic and will probably lead to long-term effects. Additionally, a systematic review about Covid impacts on children and adolescents general health indicates a significant link between social isolation and anxiety and depression in Oceania, Asia, Europe and America (Almeida et al., 2021).

Regarding social support during the first quarter of 2022, Statistics Canada measured it through a single question: “How would you characterize your sense of belonging to your local community?” Participants indicated a very strong or somewhat strong sense of belonging at 47.1%, while 37.7% reported a somewhat weak or very weak sense, and 15.2% had no opinion. During the first quarter, it was observed that the sense of belonging for men aged 65 and over was 56.7%, which is higher than that for women in the same age group, at 53.1%. In the second quarter, data for the whole population was similar to the previous quarter, with the exception that 1% of respondents shifted from a strong or somewhat strong sense to a somewhat weak or very weak one. In short, the sense of belonging tends to strengthen with age. The score for the LGBTQ2+ community was the lowest in terms of the sense of belonging, with 39.6% of individuals in this subgroup expressing a very strong or somewhat strong sense of belonging. Again, men of this sub group showed a higher score (42.8%) than women (37.6%) for the first quarter of 2022.

During the pandemic, the cultural sector, which includes musical artists, was among those most impacted with cessation of gatherings for cultural activities as reported by Canadian Artists and Content Creators Economic Survey Report (CACCES) (Statistics Canada, 2024). This report included 4,747 valid responses. The pandemic exacerbated challenges that artists already faced because of the precarity associated with gig work, self-employment and the rising costs of living. These concerns were also noted in two British studies (Cohen and Ginsborg, 2021; Spiro et al., 2021). One study involving semi structured interviews with 24 freelance musicians highlighted the significant emotional toll experienced by performing artists, including the loss of their careers, the absence of music-making opportunities and connections with colleagues, and concerns about the future of the music industry (Cohen and Ginsborg, 2021). In a separate study, researchers examined the effects of COVID-19 on the well-being of 385 performing artists by administering standardized psychology questionnaires. As anticipated, financial difficulties correlated with decreased well-being and increased depression and loneliness scores. Notably, individuals who engaged in more physical activity before a lockdown exhibited higher levels of well-being and social connectedness, along with lower loneliness scores. Furthermore, an uptick in physical activity during lockdowns, coupled with older age, was associated with elevated levels of well-being and social connectedness, as well as decreased depression and loneliness scores (Spiro et al., 2021).

Even before the pandemic, research has shown that music-making could impact the well-being of musicians at various stages in their development and was not just associated with professional musicians. For example, as part of a comparative case study, Jones (2018) looked at the choral experience of six non-music majors in university settings and found that music-making contributed to finding a healthy life-balance. In a study involving 375 participants engaged in various leisure activities, Stewart and Lonsdale (2016) suggest that singing in a group (choral singing) may have a greater impact on psychological well-being than singing solo. A large-scale study was conducted on 1,513 adult singers in Spain using a measurement tool to determine potential benefits of choral singing (Fernández-Herranz et al., 2022). The measure comprised five components (satisfaction, ability, group engagement, belonging, and optimism) and results indicated that choral singing impacted well-being. Lonsdale and Day (2021) conducted a study with 194 respondents in which they compared levels of well-being in six types of activity (choir singers, solo singers, band/orchestra members, solo musicians, team sport players, and solo sport players). They found that while choral membership offered psychological benefits, these were not unique to choir itself and that other individual and group activities in music and sports that promote self-accomplishment could provide similar benefits.

Studies indicate that music-making can impact mental health both positively and negatively. For example, one study explored the choral experiences of 21 adult amateur choir members, many of whom suffered from health issues, including long-standing mental health challenges, physical and intellectual disabilities. Based on interviews conducted at three points during a 12-month period, researchers found evidence that choral singing impacted participants on a personal level (e.g., emotional regulation), social level (e.g., social functioning) and through functional outcomes such as employment capacity (Dingle et al., 2013). Stress relief has been a noted benefit of music-making for musicians in high school ensembles (Varner, 2017) and in community settings (Mantie, 2013). Conversely, ensemble music-making has also been found to have negative impacts on mental health, including anxiety, social phobia, and depression of 377 musicians in professional orchestras (Kenny et al., 2014) and on anxiety of 201 semi-professional choral singers (Ryan and Andrews, 2009). It seems that some professional musicians may experience more anxiety or depression than non-musicians, especially if they have a prominent role, such as a soloist (Vaag et al., 2016) and younger professional musicians suffer from anxiety more than older professional musicians (Kenny et al., 2014).

The social nature of music ensembles is an aspect that is often appreciated by musicians of all ages and one that also offers social benefits. For example, a study on 72 adolescent orchestral and concert band musicians found that they favorably viewed ensemble participation as opportunities to meet new people and spend time with others, and participants highly rated these social aspects when considering continued membership (Hewitt and Allan, 2013). Similar findings were found in studies with older adults (Creech et al., 2014; Barbeau and Cossette, 2019; Barbeau and Mantie, 2019). Another study conducted with 10 small choirs ranging from 20 to 80 participants and a large choir with 232 participants demonstrated that the effects on well-being were positively similar, but that a greater sense of belonging was reported by participants in the larger choir (Weinstein et al., 2016).

Studies have shown that choirs are also a place for creating social connection and developing social relationships (Cohen, 2012; Dingle et al., 2013; Lamont et al., 2018). The social aspect of ensemble music making can promote group cohesion and provide motivation for young people to join and stay in a group (Adderley et al., 2003; Matthews and Kitsantas, 2007; Mantie, 2013). In a study on band, choral and orchestra ensembles, 60 high school students indicated they valued their ensemble relationships and were motivated to participate for both social and musical benefits (Adderley et al., 2003). In a separate study comparing 660 young musicians with 655 non-musicians, results revealed that those who participated in group music-making further developed students’ socioemotional competencies and leadership skills than those who did not (Ros-Morente et al., 2019).

The aim of the study was to investigate the effects of musical practice on well-being, mental health and social support among Canadian musicians aged 14 years old and over during the first wave of COVID 19 up to the first half of 2022. The objectives of the study were to study whether factors such as age, gender, sports, hobbies, volunteer work, frequency of musical practice, music level and types of practice were associated with psychosocial changes in musicians. Our hypotheses were that among participants who engaged with music during the pandemic: (1) older musicians and male musicians would have the highest levels of well-being, mental health and social support; (2) participation in group activities (sports, hobbies, volunteer work) would lead to beneficial psychosocial effects; (3) increased frequency of musical practice would positively impact well-being, mental health and social support; (4) amateur musicians would report stronger psychosocial benefits than musicians of other levels (middle/high school, post-secondary, professional); and (5) the types of practice would not affect well-being and mental health, but participation in group music-making (vocal, instrumental, or mixed ensemble) would be associated with higher social support than engagement in individual practice (solo or electronic music).

## 2 Materials and methods

### 2.1 Participants

This study was part of a larger project on the effects of music on well-being, mental health and social support in Canada at the time of the pandemic, which included populations of musicians and non-musicians, and investigated elements such as participants’ music listening habits, music learning opportunities while growing up, and perceptions about the effect of the pandemic on their life. Inclusion criteria included: (1) to be 14 years or older and (2) to have Canadian citizenship or be a resident in Canada at the time of the survey. For the current study, a third criteria was added: to be a musician or to have a musical practice. Participants were recruited through networks of Canadian music associations (e.g., the Coalition for Music Education in Canada, the Canadian Band Association) professional and community conductors, university professors in music education, high school music teachers, and social media. This study was approved by the ethics Board of the Université du Québec à Montréal.

A sample of 2,438 Canadians completed the survey. In order to determine the third inclusion criteria, we asked participants if they self-identified as musicians, and later in the survey, to select whether or not practicing music was part of the activities with which they currently engaged. Since we were primarily interested in understanding the effect of musical engagement on psychosocial health, we decided to include only participants who declared having a musical practice in the following analyses. As a result, 1,619 participants (66.41%) were classified with having a musical practice and 819 without (33.59%). The current study was only carried out with the 1,619 respondents categorized as musicians (be they students, amateurs, or professionals).

### 2.2 Instrumentation

The online survey was developed by the authors using the LimeSurvey platform. It included demographic information (such as age, gender, ethnicity primary occupation, socioeconomic status, etc.), general questions (about hobbies, groups activities, music listening habits, etc.), music-related questions (about instruments played, musical level, frequency of practice, etc.), as well as three standardized and psychometrically sound questionnaires, all recommended by the *Institut national de santé public du Québec*, a public health expertise and reference center (Canuel et al., 2020):

- The WHO-5 is a measure that assesses well-being in the last two weeks, through questions related to mood (feeling cheerful, calm, energetic, well-rested) and interests in a person’s daily life. It is an effective and often used screening tool that shows appropriate psychometric properties (Topp et al., 2015). This instrument, in the form of a comprehensible and short questionnaire, has been validated to evaluate subjective and psychological wellbeing and consists of five positively-worded items (Henkel et al., 2003; Bech, 2004) that can be answered on a 6-point scale ranging from 0 (At no time) to 5 (All of the time). The maximum score is 25, which can be transformed into a 0–100 scale by multiplying the score by four. Higher scores indicate a better state of wellbeing. Studies with general populations estimate that the average score generally falls around 70 (Topp et al., 2015). Its use is appropriate for participants from 9  years of age onwards (Allgaier et al., 2012, 2013).
- The MHC-SF is a tool that evaluates positive mental health (Keyes, 2002), demonstrates acceptable psychometric properties for both adults and adolescents (Lamers et al., 2011), and has been validated with a Canadian population (Orpana et al., 2017). The instrument consists of 14 items and considers three aspects: emotional, psychological and social well-being. Answers are provided using a 6-point scale of frequency ranging from 0 (never) to 5 (everyday), with a maximum total score of 70. Classification of responses includes flourishing mental health (higher score), moderate mental health, or languishing mental health (lower score). The questionnaire is recognized by both the Canadian and Quebec governments and is used in official statistical surveys (Gilmour, 2014; Orpana et al., 2017).
- The SPS-10 measures social support through five subscales: emotional support or attachment, social integration, reassurance of worth, tangible help, and orientation. This 10-item questionnaire uses a four-point Likert-type scale ranging from 1 (strongly agree) to 4 (strongly disagree). Scores may vary from 10 to a maximum of 40. Because no neutral value is available on that scale, participants are obligated to position themselves (either agreeing or disagreeing) on each item. It is important to note that all items are worded positively, which means that a lower score indicates higher social support. This tool has been validated among various populations and shows adequate psychometric properties (Cutrona and Russell, 1987; Caron, 2013).

The complete survey is available in the Supplementary material.

### 2.3 Data analysis

Descriptive statistics were undertaken. Survey data were analyzed through statistical testing (mainly multiple linear regressions) and were performed with the R software. The distributions of the three dependent variables were checked and they all followed a normal distribution which is an assumption of the multiple linear regression. All the other assumptions were checked using the residuals of the regressions. A range of independent variables were selected to assess their effects on levels of well-being, mental health and social support. More specifically, we examined whether age (continuous variable), sex (male/female/non-binary), participation in group activities, as well as participants’ musical level, frequency of musical practice, and types of musical practice could influence the three dependent variables. It is worth noting that the types of group activities in which participants were involved were categorized as follows: music practice, sports, hobbies (including social clubs, such as The Lion’s Club, Rotary clubs, reading groups, chess clubs, etc.; and artistic hobbies, such as theater groups, dance groups and visual arts clubs), and volunteer work. Self-identified musical levels comprised four categories: (1) middle or high school students (including school-aged children and adolescents having access to music through formal channels such as school, private music lessons, after-school activities, etc.); (2) post-secondary students (including undergraduate or graduate music students and/or musicians engaging in music at an advanced level without identifying as professional); (3) amateur/community musicians (adults engaging in music as a form of hobby) and (4) professional musicians (adults performing at a high level of mastery, generally being paid for music performances). Frequency of musical practice was classified in five categories: every day, few times a week, once a week, few times a month, and once a month or less. Types of musical practice included solo, vocal ensemble, instrumental ensemble, mixed ensemble (vocal and instrumental), and electronic/digital music.

## 3 Results

### 3.1 Descriptive statistics

#### 3.1.1 Demographic information

The average age of participants was 36.8 years old (SD = 18.4), with a span ranging from 14 years old to 92 years old. In terms of gender, 58.7% of participants characterized themselves as female (*n* = 940), 38.7% as male (*n* = 619) and 2.62% as non-binary (*n* = 42). Eighteen musicians preferred not to disclose their gender. In terms of ethnicity, participants were mostly of European descent (*n* = 1,222; 76%), followed distantly by respondents of East and South-East Asian descent (*n* = 147; 9.14%), multi ethnic descent (*n* = 44; 2.74%), African descent (*n* = 42; 2.61%), West Asian descent (*n* = 33; 2.05%), Latin American descent (*n* = 30; 1.87%), Indigenous descent (*n* = 20; 1.24%), and Middle Eastern descent (*n* = 19; 1.18%). Some individuals specified an “other descent” (*n* = 14, 0.87%) such as “American indigenous (Cherokee),” “Uzbek,” “Jewish,” “Canadian.” Thirty-seven participants (2.3%) indicated “I do not know” or “prefer not to answer.”

Participants were spread throughout Canada, although the majority were located in the provinces of Ontario (*n* = 581 or 35.9%) and Quebec (*n* = 355 or 21.9%). No musician located in the Yukon was recruited. Table 1 summarizes the distribution of participants in comparison with the proportion of population estimated by Statistics Canada in the first half of 2022 (Statistics Canada, 2023a).

**Table 1:** Distribution of musicians by provinces in comparison with the general population.

Provinces and territories	Participants		Canadian population	
	*n*	*%*	*n*	*%*
Ontario	581	35.9	14,996,014	38.9
Quebec	355	21.9	8,650,692	22.3
British Columbia	165	10.2	5,273,809	13.73
Manitoba	131	8.09	1,405,197	3.63
Alberta	122	7.54	4,480,956	11.58
Nova Scotia	70	4.32	1,014,827	2.62
Newfoundland and Labrador	67	4.14	52,924	0.14
New Brunswick	35	2.16	801,778	2.07
Prince Edward Island	33	2.04	16,552	0.04
Saskatchewan	30	1.85	1,173,366	3.03
Northwest Territories	26	1.61	44,828	0.12
Nunavut	4	0.25	40,489	0.10
Yukon	0	0	43,454	0.11
Total (*N*)	1,619	100	38,683,567	100

#### 3.1.2 Music role

We asked participants to identify their musical role(s). Most participants identified themselves as having a single role (*n* = 907; 56%). Others selected multiple roles (*n* = 691; 42.7%), while some (*n* = 21; 1.3%) did not select any role from the list provided. Overall, people selected between zero and five roles (*M* = 1.69; SD = 0.98), among which being a “music performer” was the most common (*n* = 1,287; 79.5%), followed by being a “music teacher” (*n* = 625; 38.6%), “conductor” (*n* = 297; 17.2%), and “composer” (*n* = 202; 12.5%). Other roles (e.g., sound recorder, arranger, producer, music therapist, arts administrator) were selected by 200 respondents (12.4%).

#### 3.1.3 Instrument played

**Figure 1:**
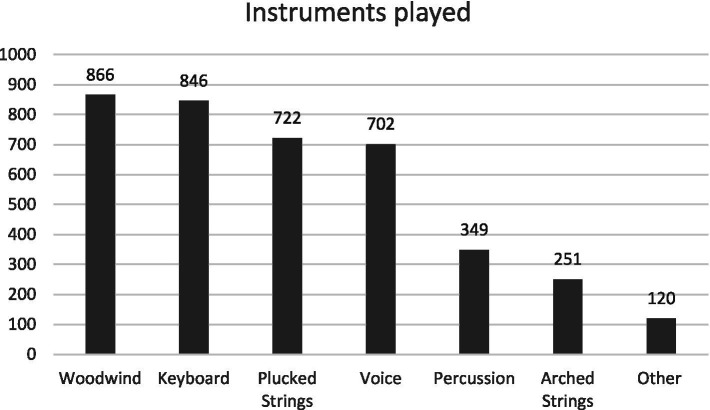
Descriptive statistics of instrument played (by family of instruments). Examples of “other” instruments included organ, kalimba, bagpipe, handbells, harpsichord, theremin, daxophone, and otamatone. Participants (*N* = 1,619) could select multiple options.

Participants reported on the instruments they played from a list provided. We regrouped these instruments by family (Figure 1). Participants played up to 23 instruments (*M* = 3.66, *SD* = 3.07). A total of 1,258 participants (77.7%) played multiple instruments and 359 (22.2%) a single instrument. Examples of instruments provided by respondents in the “Other” category included organ, kalimba, bagpipe, handbells, harpsichord, theremin, daxophone, and otamatone. Two participants (0.1%) did not select an instrument from the list, nor did they specify one in the category “Other.”

#### 3.1.4 Level, frequency and types of musical practice

**Figure 2:**
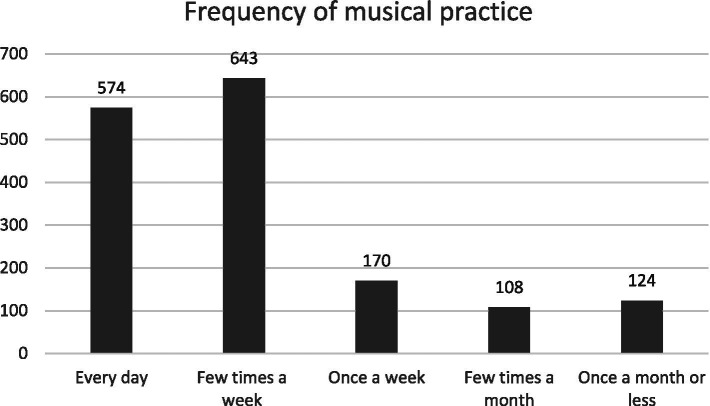
Descriptive statistics of the frequency of musical practice (*N* = 1,619).

The average years spent playing music was 18.1 (*SD* = 10.6), with a range from one to 30 years. Self-reported level of musical practice was mostly equally spread among the sample (Table 2), and the vast majority of participants (75%) played or sang at least a few times a week if not every day (Figure 2).

**Table 2:** Level of musical practice.

Level of musical practice	Overall	
	*n*	%
Middle or High school student	384	23.7
Post-Secondary level (college or conservatory, university)	480	29.6
Amateur/Community music	443	27.3
Professional level	312	19.3
Total (*N*)	1,619	100

Most musicians (*n* = 1,172; 72.4%) reported practicing music in solo, whereas in terms of group music-making, instrumental ensembles was the type of practice that was endorsed by the most musicians, i.e., 64.5% of participants (*n* = 1,045). Because participants could choose multiple options, other forms of musical practice were also provided. Results showed that among the sample, 490 respondents (30.5%) were involved n mixed ensembles (combining vocalists and instrumentalists), 423 (26.1%) in vocal ensembles, and 196 (12.1%) in electronic/digital music.

Participants played music mainly in the community (*n* = 783; 48.4%) and with friends (*n* = 655; 40.5%). Several respondents also indicated being involved in music in an educational environment, such as in schools (*n* = 491; 30.1%) and in after-school programs (*n* = 305; 18.8%), while some specified that they did not play music in a group (*n* = 136; 8.4%).

#### 3.1.5 Sports, hobbies and volunteer work

In this section, we asked questions from the general to the specific. We started by exploring if participants had hobbies (to which 98.7% answered positively) and if they participated in group activities (to which 89.3% said yes). Then, we categorized group activities and found that our sample was mostly involved in sports and volunteer work, followed by social clubs and artistic hobbies (Table 3).

**Table 3:** Sports, hobbies, and volunteer work.

Types of groups activities*	Overall	
	*n*	%
Sports	610	37.7
Volunteer work	606	37.4
Social clubs	515	31.8
Artistic hobbies (Theatre, dance, visual arts clubs)	347	21.4
Other hobbies (e.g., Gaming, gardening, circus, riding, playing bridge)	106	6.5

*Participants (*N* = 1,619) could select multiple options.

### 3.2 Multiple linear regression

The average score of the 1,619 participants on the WHO-5 was 56.9 (SD = 20.6), on the MHC-SF was 44.2 (SD = 13.7), and on the SPS-10 was 16.1 (SD = 5.63).

#### 3.2.1 Age and gender

Age was found to be a significant predictor of well-being (*b* = 0.16, *β* = 0.14, *p* < 0.001), mental health (*b* = 0.14, *β* = 0.18, *p* < 0.001), and social support (*b* = −0.03, *β* = −0.09, *p* < 0.001), which means that with increasing age, scores on the WHO-5, MHC-SF, and SPS-10 significantly improved.

When compared by gender (Table 4), there was a significant difference between males and females on the WHO-5 (*b* = −2.20, *β* = −0.11, *p* = 0.044) as well as between males and non-binary individuals (*b* = −11.77, *β* = −0.57, *p* < 0.001). Post-hoc analysis also showed that non-binary participants reported significantly lower scores than female participants [*t*(1491) = 2.93, *p* = 0.01]. Regarding mental health, males and females reported similar mean scores [*t*(1491) = 1.113, *p* > 0.05], while the average score of non-binary participants significantly differed than the ones of males [*t*(1491) = 5.447, *p* < 0.001] and females [*t*(1491) = 5.142, *p* < 0.001]. In terms of social support, it was found that male participants reported a significantly higher mean score than female participants (*b* = −0.83, *β* = −0.15, *p* < 0.004), indicating that they experienced less social support. While non-binary individuals experienced the highest SPS-10 scores, it was not found to reach statistical significance when compared with males and females’ scores, which is probably due to the smaller sample size.

**Table 4:** Average scores and standard deviations by gender.

Gender	WHO-5 (/100)		MHC-SF (/70)		SPS-10* (/40)	
	*M*	*SD*	*M*	*SD*	*M*	*SD*
Male	58.7	20.9	44.9	13.7	16.8	5.97
Female	56.4	20.2	44.5	13.4	15.6	5.35
Non-Binary	42.7	19.3	29.4	13.3	17.2	5.75

*Lower scores indicate better social support.

#### 3.2.2 Sports, hobbies and volunteer work among musicians

Results varied between 56.3 and 59.6 on the WHO-5, between 43.4 and 46.5 on the MHC-SF and between 15.2 and 16.7 on the SPS-10 (Table 5). Sports was found to be a significant predictor of well-being, mental health and social support. Indeed, musicians who practice sports reported significantly better scores on the WHO-5 (*b* = 4.13, *β* = 0.10, *p* < 0.001), MHC-SF (*b* = 3.46, *β* = 0.12, *p* < 0.001), and SPS-10 than those who did not (*b* = −0.65, *β* = −0.06, *p* = 0.021). Regarding hobbies, participants involved in social clubs reported higher scores on the WHO-5 (*b* = 4.01, *β* = 0.09, *p* = 0.001) and MHC-SF (*b* = 2.97, *β* = 0.10, *p* < 0.001) than those who did not. No significant difference was observed for social support on the SPS-10 (*b* = −0.59, *β* = −0.05, *p* = 0.058). As for participants involved in artistic hobbies such as theater groups, dance groups or visual arts clubs, no significant difference was found on wellbeing (*b* = 0.65, *β* = 0.01, *p* = 0.617), mental health (*b* = −0.28, *β* = −0.01, *p* = 0.741), and social support (*b* = 0.45, *β* = 0.03, *p* = 0.177). Volunteer work was a significant predictor of mental health (*b* = 1.50, *β* = 0.05, *p* = 0.040) and social support (*b* = −1.05, *β* = −0.09, *p* < 0.001), but not wellbeing (*b* = 1.35, *β* = 0.03, *p* = 0.233). 

**Table 5:** Average scores and standard deviations by types of group activity.

Group activities	WHO-5 (/100)		MHC-SF (/70)		SPS-10* (/40)	
	*M*	*SD*	*M*	*SD*	*M*	*SD*
Sports	59.6	19.4	46.5	13.7	15.6	5.46
Social clubs	57.9	21.2	45.1	13.4	16.0	5.55
Artistic hobbies (Theater, dance, visual arts)	56.3	21.9	43.4	13.8	16.7	5.50
Volunteer work	57.8	20.8	45.6	14.4	15.2	5.09

*Lower scores indicate better social support.

#### 3.2.3 Frequency of musical practice

Results on the WHO-5 ranged from 52.5 and 58, from 38.9 to 45.9 on the MHC-SF, and from 15.2 to 17.4 on the SPS-10 (Table 6). For the regression analysis, frequency of musical practice was considered a continuous variable. Results showed that well-being tended to increase with the frequency of musical practice (*b* = 0.97, *β* = 0.06, *p* = 0.039). For instance, people who practiced everyday had higher WHO-5 scores than people who practiced less often. There was also a positive effect on mental health: increasing the frequency of practice tended to significantly increase MHC-SF scores (*b* = 1.16, *β* = 0.10, *p* < 0.001). No significant association was found between the frequency of musical practice and social support (*b* = −0.10, *β* = −0.02, *p* = 0.435).

**Table 6:** Average scores and standard deviations by frequency of musical practice.

Frequency of musical practice	WHO-5 (/100)		MHC-SF (/70)		SPS-10* (/40)	
	*M*	*SD*	*M*	*SD*	*M*	*SD*
Every day	57.6	20.4	45.9	13.5	15.2	5.08
Few times a week	58.0	20.2	44.6	13.2	16.2	5.91
Once a week	55.8	19.6	42.5	13.2	16.9	5.53
Few times a month	52.5	23.2	41.1	15.4	17.4	5.40
Once a month or less	53.4	21.4	38.9	14.9	17.4	6.27

* Lower scores indicate better social support.

#### 3.2.4 Music level

When compared by music level, scores on the questionnaires varied slightly (Table 7). Amateur musicians were found to have significantly higher WHO-5 scores than professional musicians (*b* = −4.39, *β* = −0.21, *p* = 0.007) and respondents doing music at a post-secondary level (*b* = −3.37, *β* = −0.16, *p* = 0.02). No other comparisons reached statistical significance.

**Table 7:** Average scores and standard deviations by music level.

Music level	WHO-5 (/100)		MHC-SF (/70)		SPS-10* (/40)	
	*M*	*SD*	*M*	*SD*	*M*	*SD*
Amateur	60.5	20.8	46.6	13.2	16.1	5.91
Secondary	54.8	20.3	40.3	14.6	17.2	5.21
Post-secondary	56.2	20.1	44.5	13.2	16.0	5.86
Professional	55.4	20.8	45.1	13.2	14.9	5.10

*Lower scores indicate better social support.

Regarding mental health, the only significant result was that amateur musicians had higher MHC-SF scores than professional musicians (*b* = −2.08, *β* = −0.15, *p* = 0.047). In terms of social provision, no significant difference was detected between music levels.

It is worth noting that while secondary students reported the lowest WHO-5 scores and MHC-SF scores and the highest SPS-10 scores in the music level category, results on the regressions were not found to be statistically significant for this group when compared to our reference group (amateurs). This result will be addressed in the discussion.

#### 3.2.5 Types of practice

Mean scores slightly varied between types of practice (Table 8). It is worth noting that because participants could select more than one type of musical practice (solo, vocal, instrumental, mixed ensemble, electronic music), comparisons were made between participants involved in each type of practice and those who were not. Results on the WHO-5 showed no significant difference between participants who were involved in a specific type of practice (be it solo, vocal ensemble, instrumental ensemble, mixed ensemble or electronic music) and those who were not (Supplementary Table S1). Scores on the MHC-SF were found to be significantly lower for participants having a solo musical practice (*b* = −1.58, *β* = −0.05, *p* = 0.046) than for participants who did not. No other significant differences were observed, which means that apart from solo practice, the type of musical practice in which musicians are involved does not significantly affect their mental health (Supplementary Table S2). Finally, it was found that social support was significantly better for participants having a solo practice (*b* = −0.76, *β* = −0.06, *p* = 0.016) or for those practicing music in an instrumental ensemble (*b* = −0.89, *β* = −0.08, *p* = 0.002) than for musicians not involved in these activities. No other difference was found regarding vocal practice, mixed ensemble or electronic music (Supplementary Table S3).

**Table 8:** Average scores and standard deviations by types of practice.

Type of practice	WHO-5 (/100)		MHC-SF (/70)		SPS-10* (/40)	
	*M*	*SD*	*M*	*SD*	*M*	*SD*
Solo	56.4	20.3	43.9	13.8	15.6	5.41
Vocal	59.0	20.3	45.8	12.7	16.2	5.59
Instrumental	56.5	20.0	44.0	13.9	15.7	5.47
Mixed ensemble	56.9	20.5	45.6	13.2	15.4	5.35
Electronic music	56.1	23.0	43.3	14.3	15.9	5.31

*Lower scores indicate better social support.

## 4 Discussion

The aim of the study was to better understand the effect of musical practice in terms of well-being, mental health and social support of Canadian musicians, and to examine whether other factors (i.e., gender, age) may have played a role, in a pandemic context.

The average score on the WHO-5 was 56.9 out of 100 for the whole sample, whereas the mean score in the general population is usually around 70 (Topp et al., 2015). Our sample thus seemed to be on the lower side of the spectrum in terms of well-being, although this is unsurprising, given the pandemic context. Scores on the MHC-SF was 44.2 out of 70, which may be considered high enough to be categorized as a “flourishing mental health” (Gilmour, 2014). Results on the SPS-10 was 16.1 out of 40, which is quite positive as the minimum possible score is 10 (Canuel et al., 2020) and lower scores indicate better social support.

### 4.1 Influential factors

Our first hypothesis was that both older musicians and male musicians would score the highest on all three variables. This proved to be the case for older musicians. In fact, one of the first factors that was found to have an impact on well-being, mental health and social support was the age of respondents. As age increases, scores on all three questionnaires (WHO-5, MHC-SF and SPS-10) showed a significant positive trend. These results are consistent with Statistics Canada data on well-being (2021–2022) and mental health (2022). Gender was also an influential factor, but did not meet the expectation stated in our hypothesis. While significant differences were found between males, females and non-binary individuals regarding their wellbeing (with the former reporting the highest level and the latter the lowest), mental health differed slightly: only the non-binary participants experienced significantly different (and lower) levels of mental health, when compared to males and females. Regarding social support, males were found to be significantly less advantaged than females. This last result differs from Statistic Canada, where women reported less social support than men. This may be due to the difference in data collection measures. We used the more comprehensive Social Provisions Scale (Cutrona and Russell, 1987; Caron, 2013), which consists of 10 items to assess social support, whereas Statistics Canada used a single question to assess participants’ sense of belonging to the local community.

Our second hypothesis was that participating in group activities would lead to positive effects on all variables. Results varied depending on the type of activity. Sport, for instance, was identified as a significant predictor of well-being, mental health, and social support for our participants, whereas artistic hobbies were not. In fact, musicians engaging in sports reported notably higher scores on the WHO-5, MHC-SF, and SPS-10 than those who did not. This is aligned with results from Spiro et al. (2021), which indicate that British performing artists engaged in sports activities were less impacted by the pandemic than those who did not. Findings are consistent with well-documented benefits of sports reported in the pre-pandemic literature. For instance, sport participation has been shown to reduce depression and suicidal thoughts (Babiss et al., 2009). A systematic literature review done by Eigenschenk et al. (2019) also demonstrated that sport, especially outdoor sport and recreation, had a positive impact on mood, and could decrease stress, anxiety, depression and even loneliness. However, similarly to what was found with professional musicians, it is noteworthy that elite or semi-elite athletes do not benefit from sports engagement like amateurs. A meta-analysis comprising 60 studies on elite or semi-elite athletes revealed that factors such as overtraining, injuries, pressure, and the distinctive experiences encountered by athletes seem to influence the potential benefits derived from engaging in sports (Rice et al., 2016).

Participants actively involved in social clubs demonstrated higher scores on the WHO-5 and MHC-SF compared to those who were not. This aligns with existing literature. As an illustration, participation in social clubs among 1,963 middle-aged women from the USA affiliated with the Red Hat Society demonstrated a positive effect on well-being (Son et al., 2010). While similar positive effects were also observed for volunteer work, as shown in a longitudinal study based on 66,343 observations (person-years) in the UK (Tabassum et al., 2016), our findings showed that volunteer work was a significant predictor of mental health and social support, but not wellbeing. Having said that, the beneficial effects reported by Tabassum et al. (2016) are particularly evident in their sample of participants aged 40 to 70 years old. This aligns with our findings associated with age: the older our participants, the higher their scores on the three questionnaires. This is also consistent with the trends observed on well-being in the Canadian population as reported by *Statistics Canada* (Statistics Canada, 2023c).

### 4.2 Well-being, mental health and social support

Our third hypothesis was that practicing music more often would positively impact well-being, mental health and social support. Findings from our study revealed that well-being and mental health generally improved with increased frequency of musical practice (see Table 6), whereas this was not the case for social support. This aligns with other studies on well-being and choristers (Lonsdale and Day, 2021; Fernández-Herranz et al., 2022). In fact, regarding the social impact of musical engagement, Lonsdale and Day (2021) found that choral singing generated levels of well-being that were as similarly high as engaging in other types of hobbies, no matter if they were carried out in groups (choral singers, band/orchestra members, team sports players) or not (solo singers, solo instrumentalists, individual sports players).

While music can be experienced as a relaxing hobby for amateurs, research shows that professional musicians often face performance anxiety (Kenny et al., 2014), depression, and social phobia (Ryan and Andrews, 2009). Therefore, our fourth hypothesis was that amateur musicians would experience better well-being, mental health and social support than musicians of other levels. Results of subgroups presented in Table 7 show that, in line with our hypothesis, amateur musicians demonstrated significantly higher WHO-5 scores than professionals and respondents with a post-secondary level of music, as well as significantly better MHC-SF scores than professional musicians. However, no difference in social support was observed between the subgroups.

An important consideration must be taken into account for professional musicians: the inability to practice their profession during the COVID-19 pandemic. As shown in the literature, this has produced financial challenges and stress that contributed to lower levels of well-being and mental health (Cohen and Ginsborg, 2021; Spiro et al., 2021). Professional musicians, who are often freelancers, were severely affected by the cancellation of music activities. As the CACCES survey (2022) specified, 83.1% of the 863 participants who identified themselves as musical artists, which included vocalists and instrumentalists (*n* = 774), songwriters (*n* = 336), and others (*n* = 176), reported a decrease in income (the average across all fields being 62.2%). In comparison, writers experienced a 44.7% decrease. Notably, the impacts were higher for men (67%) than women (60.7%) and non-binary individuals (55.3%). Another subgroup, secondary students, reported lower scores on well-being and mental health. This could be attributed to school lockdowns lasting from 8 to 19 weeks, depending on the area. Moreover, when schools reopened, music activities such as instrumental and vocal ensembles in high schools were not (Morin, 2021; Barbeau et al., 2023). This also echoes Statistics Canada’s survey on Quality of Life, in which younger respondents (15–24 years) reported the lowest scores on mental health and well-being in 2022. For this population, Almeida et al. (2021) suggest that it is crucial for healthcare professionals to provide diligent follow-up care post-pandemic. Garagiola et al. (2022) recommend future research that will evaluate the long-term impact of Covid on adolescent development.

Regarding the types of music practice, we found that participants tended to engage in different forms of music-making. Because respondents could choose multiple options (solo, instrumental ensemble, mixed ensemble, vocal ensemble, or electronic/digital music), we compared categories separately, analyzing between musicians who engaged in each specific type of practice and those who did not. Using this strategy to examine scores on our three main variables, we found that the type of practice did not seem to have an impact on participants’ well-being, which is in keeping with our fifth hypothesis. However, results varied regarding mental health and social support. In terms of mental health, solo practice showed the lowest scores. Results from the CACCES survey (2022) may shed light on our findings. The survey showed that 89.7% of professional musicians tended to have an instrumental or vocal practice, suggesting that they may have engaged in solo practice time, at least in preparation for performances. It is possible that, in our survey, professional musicians may have been overrepresented in the category “solo,” and because they were greatly affected by the pandemic, this may have influenced our results. This is aligned with some findings reported in the literature about professional musicians (Cohen and Ginsborg, 2021; Spiro et al., 2021). In addition, as Vaag et al. (2016) indicate, professional musicians, particularly those in prominent roles like soloists, may be more prone to experiencing anxiety or depression compared to non-musicians. Interestingly, social support results were found to favor respondents with a solo practice and those practicing music in an instrumental ensemble. While it seems evident why practicing music in a group, such as an instrumental ensemble, may have an impact on social support, it is not clear why this may be the case for solo practice. Could it be that people who reported having a solo practice were also simultaneously engaged in other forms of group music-making, which may have skewed the results? Could it be that when the lock-downs happened, all musicians had to stop playing music in groups but found comfort in knowing that all of them could continue to do music at home, and even produce collective works online? Levstek et al. (2021) results showed the benefits of virtual group music making for children and young people. Could this be the same for the general population? There are no definite answers.

### 4.3 Limitations and future directions

When comparing the proportion of population estimated by Statistics Canada in the first half of 2022 (Statistics Canada, 2023d) with our sample size, we found that there was an over-representation of respondents in the provinces of Manitoba (over 5%), Nova Scotia (over 2%), Newfoundland/Labrador (4%), Prince Edward Island (over 2%), and Northwest Territories (near +1.5%), and an under-representation in Ontario (−3%), British Columbia (near-3%), Alberta (near −4%), and Saskatchewan (near −1.5%). Because the population is distributed very differently between the provinces and territories, a difference in number may impact the representativeness of the population significantly more in the Northwest Territories than in Ontario, for instance. Also, the cultural reality of the inhabitants of a large city in a French-speaking province like Quebec is very different from that of an English-speaking town in Alberta, especially as educational and cultural policies are decentralized. For example, we found that organizations of music professionals that could relay the surveys were practically non-existent in some locations (i.e., Yukon) or very dynamic in others (i.e., Ontario and Quebec). This may explain why survey respondents are distributed somewhat differently from the Canadian population.

To put into perspective the results of participants in our study whose gender is non-binary with those of the Canadian population, we used the LGBTQ2+ subcategory available in Statistics Canada’s data. However, this subcategory includes not only gender identity but also sexual orientation. Consequently, the comparison samples are not similar. As we did not want to exclude the results obtained from the responses of non-binary individuals from a cross-comparison with the Canadian population, we retained this comparison. In future studies, it may be beneficial to use the same subcategories as those in Statistics Canada for potential comparison.

Our recruitment process deliberately targeted instrumental musicians because there had already been a major Canadian survey conducted in 2021 by Choral Canada, which had examined the impact of the pandemic on singing in Canadian schools (Morin, 2021). As a result, we collaborated with multiple partners to reach as many instrumentalists as possible which helped us recruit a significant number of participants. However, this may have also biased the type of musicians recruited. In future studies, it would be valuable to develop recruitment strategies that target independent musicians, musicians not associated with institutions, and musicians with informal or non-Western music practice.

Regarding the questionnaire’s design, it is worth noting that the survey responses were self-reported. As such, it is possible that participants may have answered inaccurately, voluntarily or not. For instance, the level of musical practice: participants were asked to self-assess their musical proficiency, which could result in different interpretations based on individual perceptions. Originally, we had designed the questionnaire with three categories (“middle/high school student,” “amateur,” “professional”). However, we received feedback when we piloted the questionnaires that one category was missing in order to include musicians who performed music at a higher level than secondary school or amateur but did not consider themselves professional because music was not their primary source of income. This led us to add the category “post-secondary level,” which included college or university music students, but also those who graduated from post-secondary music institutions and continued to perform music (paid or not) on the side. This raised the issue about how that question was understood by respondents, which led also to a more philosophical question: what makes a musician “professional”? Is it related to income, fame, musical prowess? While the purpose of the article is not to answer that question, the fact that multiple interpretations were possible suggest that we should have defined these categories for participants in order to minimize confusion.

Secondly, regarding participants engaged in artistic hobbies such as theater groups, dance groups, or visual arts clubs, no significant difference was found in well-being, mental health, and social support. At the same time, because all these artistic hobbies were combined as part of one large category, it is impossible to determine whether results would have differed if these hobbies would have been analyzed separately. It would be worth investigating this aspect in the future.

Thirdly, like we did for music, it would have been worthwhile to collect information about the time spent doing sports, hobbies, and volunteer work, in order to better compare the scope of the results obtained in terms of number of years and hours a week of involvement in other activities. Further research is needed to examine the effects of practicing more than one type of group activity (for instance, music and sport, music and volunteer work) on well-being, mental health and social support.

Finally, it may be useful to explain the statistical analysis and how some of the results may seem counterintuitive at first glance. For instance, with non-binary participants (in our analysis by gender) or with respondents playing music at a middle/high school level (in our analysis by music level), comparisons with the reference groups did not reach statistical significance despite the fact that their average scores were the lowest of their respective category. While this could be attributed to a type II error (which means a failure to reject the null hypothesis of no difference while there may have been an actual effect; Olsen, 1987), it is more likely that the corrections that were applied for statistics involving multiple testing (which control for specific error rates; Bender and Lange, 2001) decreased the apparent significance of effects (Chen et al., 2017).

## 5 Conclusion

This study was conducted as part of a larger project on the effects of music on the well-being, mental health and social support of Canadians and involved studying student, amateur and professional musicians in Canada during the Covid-19 pandemic. Results showed that musicians with more frequent musical practice reported greater well-being and mental health, as did practicing musicians at an amateur level. Findings also demonstrated that mental health tended to be lower for musicians reporting having a solo music practice, but that social support was stronger for them and those who performed music in instrumental ensembles. Factors such as age, gender, and practicing sports were found to have a significant impact on well-being, mental health and social support. Additionally, being involved in social clubs or doing volunteer work were associated with benefits on two of the three variables (better mental health and well-being for social clubs, and better mental health and social support for volunteer work). With the above findings, we can posit that in the context of the COVID-19 pandemic, regular amateur musical practice, primarily in a group setting, coupled with sports, may have fostered well-being, mental health, and a sense of social support.

## Data availability statement

The raw data supporting the conclusions of this article will be made available by the authors, without undue reservation.

## Ethics statement

The studies involving humans were approved by Comité Institutionnel d’Éthique de la recherche avec des êtres humains (CIEREH) de l’Université du Québec à Montréal (certificate number: 2022-4,230). The studies were conducted in accordance with the local legislation and institutional requirements. The participants provided their written informed consent to participate in this study.

## Author contributions

A-KB: Writing – original draft, Writing – review & editing. IH: Writing – original draft, Writing – review & editing. GR: Writing – original draft, Writing – review & editing. L-ÉT-P: Writing – original draft, Writing – review & editing.

## Footnotes

^1^According to Statistics Canada, “This category includes people who reported their sexual orientation as lesbian, gay, bisexual, pansexual, or a sexual orientation not elsewhere classified. It also includes persons whose reported sex assigned at birth does not correspond to their gender, including those whose gender is not exclusively man or woman (regardless of sexual orientation)” (Statistics Canada, 2023c).
