# Supplementary material for: The effects of musical practice on the well-being, mental health and social support of student, amateur, and professional musicians in Canada during the COVID-19 pandemic
Source: Front Psychol. 2024 Jun 7;15:1386229. doi: 10.3389/fpsyg.2024.1386229 (PMC11192208; doi:10.3389/fpsyg.2024.1386229)
Supplement: Supplementary file 1 [file Table_1.docx]

**Supplementary Table**

**Regression analysis**

| **Supplementary Table 1**  *Results of the multiple linear regression fo WHO-5* | | | |  |
| --- | --- | --- | --- | --- |
| Predictors | *b* | β | *t* | *p* |
| (Intercept) | 47.53 | 0.12 | 12.28 | <0.001 |
| Age in years (continuous) | 0.16 | 0.14 | 4.45 | <0.001 |
| Gender = Male | Reference |  |  |  |
| Gender = Female | -2.20 | -0.11 | -2.01 | 0.044 |
| Gender = Non-Binary | -11.77 | -0.57 | -3.56 | <0.001 |
| Sports | 0.65 | 0.01 | 0.50 | <0.001 |
| Social clubs | 4.01 | 0.09 | 3.37 | 0.001 |
| Artistic hobbies  (theater, dance, visual arts) | 0.65 | 0.01 | 0.50 | 0.617 |
| Volunteer work | 1.35 | 0.03 | 1.19 | 0.233 |
| How often do you make music (continuous) | 0.97 | 0.06 | 2.06 | 0.039 |
| MusicaLevel : Amateur | Reference |  |  |  |
| MusicLevel : Secondary | -0.66 | -0.03 | -0.38 | 0.705 |
| MusicLevel : Post-Secondary | -3.37 | -0.16 | -2.33 | 0.020 |
| MusicLevel : Professional | -4.39 | -0.21 | -2.71 | 0.007 |
| Musical practice = solo | -1.64 | -0.04 | -1.34 | 0.181 |
| Musical practice = vocal ensemble | 2.24 | 0.05 | 1.83 | 0.067 |
| Musical practice = instrumental ensemble | -0.81 | -0.02 | -0.72 | 0.474 |
| Musical practice = mixed ensemble | -0.61 | -0.01 | -0.72 | 0.608 |
| Musical practice = electronic music | -0.43 | -0.01 | -0.26 | 0.794 |
| Observations | 1531 |  |  |  |
| R^2^/R^2^ adjusted | .094 / .070 |  |  |  |
