# Supplementary material for: The effects of musical practice on the well-being, mental health and social support of student, amateur, and professional musicians in Canada during the COVID-19 pandemic
Source: Front Psychol. 2024 Jun 7;15:1386229. doi: 10.3389/fpsyg.2024.1386229 (PMC11192208; doi:10.3389/fpsyg.2024.1386229)
Supplement: Supplementary file 2 [file Table_2.docx]

**Supplementary Table**

**Regression analysis**

**Supplementary Table 2**

| *Results of the multiple linear regression for the MHC-SF* | | | |  |
| --- | --- | --- | --- | --- |
| Predictors | *b* | β | *t* | *p* |
| (Intercept) | 35.13 | 0.16 | 14.00 | <0.001 |
| Age in years (continuous) | 0.14 | 0.18 | 5.89 | <0.001 |
| Gender = Male | Reference |  |  |  |
| Gender = Female | -0.79 | -0.06 | -1.11 | 0.266 |
| Gender = Non-Binary | -11.67 | -0.85 | 5.45 | <0.001 |
| Sports | 3.46 | 0.12 | 4.95 | <0.001 |
| Social Club | 2.97 | 0.10 | 3.85 | <0.001 |
| Artistic hobbies  (theater, dance, visual arts) | -0.28 | -0.01 | -0.33 | 0.741 |
| Volunteer | 1.50 | 0.05 | 2.05 | 0.040 |
| How often do you make music (continuous) | 1.16 | 0.10 | 3.79 | <0.001 |
| MusicLevel : Amateur | Reference |  |  |  |
| MusicLevel : Secondary | -1.10 | -0.08 | -0.97 | 0.330 |
| MusicLevel : Post-Secondary | -0.83 | -0.06 | -0.89 | 0.375 |
| MusicLevel : Professional | -2.08 | -0.15 | -1.99 | 0.047 |
| Musical practice = solo | -1.58 | -0.05 | -1.99 | 0.046 |
| Musical practice = vocal ensemble | 1.42 | 0.05 | 1.79 | 0.074 |
| Musical practice = instrumental ensemble | -0.73 | -0.03 | -1.00 | 0.320 |
| Musical practice = mixed ensemble | 0.93 | 0.03 | 1.21 | -.227 |
| Musical practice = electronic music | -0.61 | -0.01 | -0.57 | 0.567 |
| Observations | 1531 |  |  |  |
| R^2^/R^2^ adjusted | .154/.132 |  |  |  |
