# Supplementary material for: The effects of musical practice on the well-being, mental health and social support of student, amateur, and professional musicians in Canada during the COVID-19 pandemic
Source: Front Psychol. 2024 Jun 7;15:1386229. doi: 10.3389/fpsyg.2024.1386229 (PMC11192208; doi:10.3389/fpsyg.2024.1386229)
Supplement: Supplementary file 3 [file Table_3.docx]

**Supplementary Table**

**Regression analysis**

# Supplementary Table 3

| *Results of the multiple linear regression for the SPS-10* | | | |  |
| --- | --- | --- | --- | --- |
| Predictors | *Estimates* | β | *Statistic* | *p* |
| (Intercept) | 18.84 | -0.10 | 18.79 | <0.001 |
| Age in years (continuous) | -0.03 | -0.09 | -2.92 | 0.004 |
| Gender = Male | Reference |  |  |  |
| Gender = Female | -0.83 | -0.15 | -2.92 | 0.004 |
| Gender = Non-Binary | -0.14 | -0.03 | -0.16 | 0.869 |
| Sports | -0.65 | -0.06 | -2.31 | 0.021 |
| Social Club | -0.59 | -0.05 | -1.90 | 0.058 |
| Artistic hobbies  (theater, dance, visual arts) | 0.45 | 0.03 | 1.35 | 0.177 |
| Volunteer | -1.05 | -0.09 | -3.60 | <0.001 |
| How often do you make music (continuous) | -0.10 | -0.02 | -0.78 | 0.435 |
| MusicaLevel : Amateur | Reference |  |  |  |
| MusicLevel : Secondary | 0.21 | 0.04 | 0.47 | 0.638 |
| MusicLevel : Post-Secondary | -0.32 | -0.06 | -0.84 | 0.399 |
| MusicLevel : Professional | -0.72 | -0.13 | -1.71 | 0.087 |
| Musical practice = solo | -0.76 | -0.06 | -2.41 | 0.016 |
| Musical practice = vocal ensemble | 0.05 | 0.00 | 0.15 | 0.883 |
| Musical practice = instrumental ensemble | -0.89 | -0.08 | -3.04 | 0.002 |
| Musical practice = mixed ensemble | -0.45 | -0.04 | -1.45 | 0.147 |
| Musical practice = electronic music | -0.62 | -0.04 | -1.48 | 0.140 |
| Observations | 1531 |  |  |  |
| R^2^/R^2^ adjusted | .154/.132 |  |  |  |
