# Supplementary material for: The effects of musical practice on the well-being, mental health and social support of student, amateur, and professional musicians in Canada during the COVID-19 pandemic
Source: Front Psychol. 2024 Jun 7;15:1386229. doi: 10.3389/fpsyg.2024.1386229 (PMC11192208; doi:10.3389/fpsyg.2024.1386229)
Supplement: Supplementary file 4 [file Data_Sheet_1.pdf]

Survey questions

The Effects of Music Practice on the Well-being, Mental Health and Social Support of Student, Amateur and Professional Musicians in Canada

Language

- Choose a language: English – Français

Identification

Citizenship:

- Are you Canadian or a permanent resident? Y/N
  - ▯ If NO: Are you currently living in Canada? Y/N
    - ▯ If NO: End of questionnaire

I am...

- 18 years old or older (adult)
  - ▯ **Consent form (Adults) -** I agree to participate in this study Y/N
- 14 to 17 years old (minor)
  - ▯ **Consent form (minors) -** My parent/legal tutor has been informed and I agree to participate in this study : Y/N
- 13 years old or younger
  - ▯ End of questionnaire

Further research

Your responses to this survey are anonymous and will be kept after the end of the project. We would like to use them in other similar research projects. You are free to decline this secondary use.

☐ I agree that this data may be used in other research projects

☐ I decline that this data may be used in other research projects

I would like to receive a summary of the results of the project: Y/N
If so, please provide your name and email address: ______________________

Introduction page

The following survey will ask questions about yourself and your hobbies, as well as questions about your well being, mental health and social support. At the end, there will be a few open-ended questions about the pandemic and how it affects you.

About yourself

- Year of birth
- Gender:
  - Male
  - Female
  - Non-binary
  - Prefer not to answer
- Ethnicity
  - East and South-East Asia descent (Japan, China, Korea, Thailand, etc.)
  - West Asia descent (India, Pakistan, Bangladesh, etc.)
  - African descent (Black)
  - European descent (White)
  - Latin American descent
  - Indigenous
  - Middle Eastern descent
  - Multiethnic
  - **Others (please specify)**
  - Don't know
  - Prefer not to answer
- Principal occupation (Select all that apply)
  - Student
  - Professional (part-time or full-time)
  - Retired
  - Unemployed
  - Caregiver
- I am a :
  - Musician (student, amateur, professional, music teacher, composer, conductor, etc.)
  - Non musician

Socioeconomic status

- In what bracket is your gross household income approximately?
  - Don't know
  - Less than $19,999
  - $20,000 to $39,999
  - $40,000 to $59,999
  - $60,000 to $79,999
  - $80,000 to $ 99,999
  - $100,000 or above
  - Prefer not to answer

Localisation

- In what province or territory are you living?
    - Alberta
    - British Columbia
    - Manitoba
    - New Brunswick
    - Newfoundland and Labrador
    - Northwest Territories
    - Nova Scotia
    - Nunavut
    - Ontario
    - Prince Edward Island
    - Quebec
    - Saskatchewan
    - Yukon

- In what type of community setting are you living? *(Check with stats can: https://www.statcan.gc.ca/fra/sujets/norme/ccprr/2016/introduction)*
    - Don’t know
    - Urban (major cities with a population above 100,000 people)
    - Mid-size cities (cities with a population between 30,000 and 100,000 people)
    - Towns, small cities (towns and cities with a population between 1,000 and 30,000 people)
    - Rural (villages with a population of less than 1000 people)

- Do you live in a suburb (i.e, close to an urban center)? Y/N

Hobbies

General questions

- Do you have any hobbies? Y/N
- Do you participate in group activities? Y/N

- In which of the following activities do you participate? (click all that apply)
    - Music practice (individual or in group)
        - Conditional response to open the section on music-related questions
    - Sports
    - Theatre or Dance or Visual arts clubs
    - Social, academic or other clubs
    - Volunteer work
    - Other: (please specify)
    - Not applicable

- Do you like to listen to music? Y/N
- How often do you listen to music that you enjoy?
    - Everyday
    - A few times a week
    - About once a week
    - A few times a month
    - Once a month or less
- Did you have music lessons in elementary school as a child? Y/N
    - In middle school or high school? Y/N

Music-related questions

(Conditional section, exclusively for participants who self-identified as musicians or who indicated practicing music as a hobby)

- I am a : (select all that apply)
    - Music performer
    - Music teacher
    - Conductor
    - Composer
    - Singer songwriter
    - Other (please specify)

- What instrument(s) do you play? (select all that apply)
    - Accordion
    - Electric bass
    - Bassoon
    - Drums
    - Singing
    - Clarinet (E flat, B flat, bass, etc.)
    - Upright bass
    - French horn
    - Euphonium / Baritone
    - Recorder
    - Flute
    - Guitar
    - Harmonica
    - Oboe
    - Percussion
    - Piano / keyboards / synthesizers
    - Saxophone (soprano, alto, tenor, baritone)
    - Trombone
    - Trumpet
    - Tuba
    - Violin
    - Viola
    - Cello
    - Ukulele
    - Other: (specify)

- How would you describe the level of your musical practice:
    - Middle or High school student
    - Post-secondary level (college or conservatory, university)
    - Amateur/Community music
    - Professional level

- For how many years have you been playing music? (numbers ranging from less than a year to over 30 years)
- How often do you make music?
    - Everyday
    - A few times a week
    - About once a week
    - A few times a month
    - Once a month or less

- Where did you first learn music (select all that apply)?:
    - In my family
    - In elementary school
    - In middle or high school
    - In church
    - Through private lessons

- Please identify the types of music practice that you do (click all that apply):
    - Solo
    - Vocal ensemble
    - Instrumental ensemble
    - Mixed ensemble (vocal and instrumental)
    - Electronic/digital music

- Please specify where you play GROUP music (check all that apply):
    - In school
    - In an after-school program
    - In the community (organized ensembles)
    - With friends
    - I do not play music in group(s)

- Complete the sentence: BEFORE the pandemic, I made music… (select all that apply)
    - On my own (Y/N)
    - Virtually with others (Y/N)
    - In-person with others (Y/N)

- Complete the sentence: DURING the pandemic, I was able to continue making music...(select all that apply)
    - On my own (Y/N)
    - Virtually with others (Y/N)
    - In-person with others (Y/N)

- Complete the sentence: CURRENTLY, I make music...(select all that apply)
    - On my own (Y/N)
    - Virtually with others (Y/N)
    - In-person with others (Y/N)

Well being, Mental health and Social Support

The three validated questionnaires used in this survey, as well as studies on their psychometric properties, are accessible online.

The **World Health Organization Well-Being Index (WHO-5)** and the **Mental Health Continuum Short Form (MHC-SF)** are available here: https://www.inspq.qc.ca/en/post-disaster-mental-health-impacts-surveillance-toolkit/standardized-measurement-instruments/questionnaires/well-being#questionnaire13

The **Social Provision Scale (SPS-10)** is available here: https://www.inspq.qc.ca/en/post-disaster-mental-health-impacts-surveillance-toolkit/standardized-measurement-instruments/information-sheets-standardized-measurement-instruments-recommended/social-support

Conclusion questions

- In a few words, how are you living the pandemic? Is it difficult for you and if so, how exactly?
- Are there positive outcomes or opportunities that resulted for you (and/or your music ensemble if you are a musician) during the pandemic? (please specify)
- Considering the issues related to the pandemic, do you have any concerns regarding your hobbies (or your music practice if you are a musician) at this point and forward? Please expand in the comment box.
